# How does participation in a voluntary prize exam affect medical students’ knowledge and interest in ENT, plastic surgery, ophthalmology and dermatology?

**DOI:** 10.1186/s12909-020-02314-y

**Published:** 2020-10-27

**Authors:** Razan Nour, Kerry Jobling, Alasdair Mayer, Salma Babikir

**Affiliations:** 1 Renal Unit, Ulster Hospital, Upper Newtownards Road, Dundonald, Belfast, UK; 2 Department of Ophthalmology, Royal Victoria Infirmary, Newcastle-upon-Tyne, UK; 3 Department of Dermatology, Royal Victoria Infirmary, Newcastle-upon-Tyne, UK; 4 Department of ENT, Freeman Hospital, Newcastle-upon-Tyne, UK; 5 Department of Vascular Surgery, Freeman Hospital, Newcastle-upon-Tyne, UK

**Keywords:** Otolaryngology, Plastic surgery, Ophthalmology, Dermatology, Knowledge and interest, Undergraduate medical students, Career choice

## Abstract

**Background:**

Otolaryngology (ENT), plastic surgery, ophthalmology and dermatology are medical specialties which tend to receive less coverage in UK medical school curricula compared to larger, generalist specialties. As a result, there are fewer opportunities for medical students to learn and to cultivate an interest. There are numerous papers that report concerns about junior doctors’ ability to manage conditions within these specialties, which may jeopardise patient safety. The aim of our pilot project was to increase medical students’ interest and knowledge of ENT, plastic surgery, ophthalmology and dermatology. In addition to describing our project, we present and discuss literature on UK undergraduate education in these specialties and its impact on preparedness of junior doctors and future career choices.

**Methods:**

One hundred twelve final year medical students at Newcastle University were invited to take part in a voluntary two-part (written and clinical) exam, in which prizes could be won and all participants would receive a certificate of participation. We distributed two online surveys to the students, one administered before the exam and one afterwards. Data was collected regarding the students’ motivation for entering the prize exam and the students’ baseline interest and knowledge in these specialties before and after the prize exam. Free-text responses were collected about the students’ opinion of the project and whether participation was beneficial.

**Results:**

Sixteen students participated in the exam. There was a statistically significant increase in the students’ knowledge in ENT (*p* < 0.000), plastic surgery (*p* < 0.000), ophthalmology (*p* < 0.028) and dermatology (*p* < 0.012) after participation in the exam, but not in their interest levels. ENT was the preferred specialty of our cohort. The students reported that they found participation beneficial to their learning, particularly receiving exam feedback and explanations to exam questions.

**Conclusions:**

This pilot project was a useful intervention in increasing medical students’ knowledge in these specialties, but not in their levels of interest. It also demonstrates that medical students are willing to participate in voluntary initiatives (in their spare time) to gain more learning opportunities and that medical students value timely exam feedback to guide their revision.

**Supplementary information:**

**Supplementary information** accompanies this paper at 10.1186/s12909-020-02314-y.

## Background

Otolaryngology (ENT), plastic surgery, ophthalmology and dermatology (EPOD) are medical specialties which tend to receive less coverage in UK medical school curricula compared to larger specialties of a more generalist nature. Several reports exist in the literature that discuss the impact that minimal exposure to these specialties has on undergraduates’ knowledge, future career choice and ability to manage associated conditions as junior doctors and beyond. Furthermore, these specialities typically attract some of the highest competition ratios for entering National Specialty Training Programmes, which demand early evidence of commitment to speciality. Limited experience as an undergraduate to these specialties, with risk of delayed career decisions, can therefore pose a problem for successful entry into these programmes. To discern the extent of this problem in the UK, we reviewed some papers related to these topics.

Despite varying lengths of undergraduate ophthalmology placements, most studies found that UK medical students receive approximately 49 h, or 7 days, of teaching in ophthalmology [1–3], with suggestions that time spent on undergraduate ophthalmology education is declining [3]. The time spent on undergraduate education in ENT was found to be similar to ophthalmology. A survey of all UK medical schools highlighted that the average time spent on ENT was 3.4 days of pre-clinical education and 5 days of clinical exposure in an ENT department, however a small proportion of respondents (15.8%) had no formal exposure to ENT [4]. This study also highlighted that 65.8% of surveyed medical students would prefer to spend more time learning ENT [4]. Most of the literature on UK undergraduate dermatology teaching has addressed content and methods rather than duration of rotation, so an accurate figure has not been specified. Although the Royal College of Surgeons have produced a national undergraduate curriculum, plastic surgery is not formally taught by all medical schools as part of undergraduate surgical teaching. This may not always have been the case however - according to a survey only 13% of medical schools formally teach plastic surgery compared to 78% in the 1980s [5].

It is estimated that to meet societal demands a large proportion of medical graduates will need to become General Practitioners. Furthermore, medical graduates who specialise in General Practice will regularly treat patients with conditions from these specialties; therefore we feel strongly that it is essential to maintain a high standard of undergraduate education covering these areas. It is estimated that 20% of adult presentations and up to 50% of paediatric presentations to GP are ENT-related [6]. Between 7 and 19% of presentations to the Emergency Department and 2–5% of primary care presentations are eye complaints [1]. There are 13 million patients who present to GPs with skin complaints in England and Wales each year [7].

There are also concerns about the ability of junior doctors to handle emergency conditions within these specialties due to the lack of exposure during medical school. A 2008 survey of senior house officers in England showed that 63.9% of respondents lacked confidence in managing eye emergencies [8]. An ENT study looked at the ability of foundation doctors working in A&E to manage emergency conditions such as stridor and epistaxis. The results showed that the respondents were not aware of basic initial management steps of common ENT emergencies such as giving supplemental oxygen in stridor [9]. This is not the first study to draw attention to UK medical graduates’ lack of confidence in managing ENT problems. Powell demonstrated that there was a statistically significant difference (*p* < 0.01) in students’ levels of confidence in ENT history taking (much lower) compared to cardiovascular history taking [4]. A 2016 systematic literature review into undergraduate ENT education in the UK concluded that the majority of final year medical students and junior doctors did not feel confident in managing ENT problems [10].

One group of researchers sent out a survey to medical students at 14 UK medical schools to determine the nature of their undergraduate dermatology teaching and to obtain information about whether their undergraduate education matched the curriculum set by the British Association of Dermatologists (BAD). They found that overall students were adequately competent at manging chronic conditions but struggled to identify and manage emergency dermatological presentations. The students also struggled with basic competencies, such as prescribing emollients and writing referral letters about skin problems [11].

There is a scarcity of literature on the preparedness of medical graduates in treating plastic surgery conditions, however we think that this is unlikely to be high given the limited training medical students receive in plastic surgery.

Due to the above concerns regarding lack of opportunity in the EPOD specialities for undergraduates, the purpose of our pilot project was to create a voluntary prize exam for final year medical students covering ENT, plastic surgery, ophthalmology and dermatology. This would be led by clinical teaching fellows and the aim was to study the impact on the levels of knowledge and interest in these specialties of the participating cohort.

## Methods

One hundred twelve final year medical students at Newcastle University were invited to take part in a voluntary two-part exam with prizes. The first part of the exam was a written examination consisting of 30 single best answer (SBA) multiple choice questions. The paper examined outcomes from the national undergraduate curricula from the respective Royal Colleges of the specialties included. Questions were original clinical scenario-based questions written by clinical teaching fellows in line with the Medical Schools Council Assessment Alliance (MSCAA) standards and screened for accuracy and suitability by relevant supervising consultants.

Immediately following the written exam, all participating final year medical students were encouraged to attend a teaching session. The purpose of this teaching session was to systematically go through the answers of the written paper and provide teaching on important topics included within the exam, highlight key learning points and give useful direction for written exam technique.

Subsequently, the four highest scoring students from the written exam entered a practical exam, in which they were required to assess real patients with conditions from the specialties involved and to perform practical tasks such as suturing. Due to this pilot project having limited capacity in terms of examiners and patient volunteers, it was only possible to permit the top 4 students to enter the practical exam. Furthermore, adding an element of competition to enter the practical exam could serve as a ‘carrot’ to motivate students’ to revise well for the exam.

The structure of the practical stations, in terms of content and timings, closely resembled the practical final exams offered at Newcastle University, known as MOSLERs (Modified Objective Structured Long Examination Review). The reason for simulating real exam conditions was to maintain consistency and avoid confusion for students facing their upcoming final exams.

Following the practical exam, the 4 finalists received constructive feedback for improvement regarding their exam performance. Sixteen students entered the written prize exam, of which the 4 highest scoring students entered the practical exam. Following the written and practical exam, a second SurveyMonkey questionnaire was administered to all 16 participants. All competing students received a certificate of participation and prizes were awarded to the finalists.

To understand whether this intervention achieved the intended aim, data was collected regarding the students’ baseline interest and knowledge in these specialties before and after participation in the Prize exam and information about the students’ motivation for entering the prize exam. This data was collected via two 10-question online questionnaires (Survey Monkey), which were sent out 2 months before participation and 2 days after the exam. These questionnaires were designed for the purpose of this project (see supplementary information).

In the first survey, the participants were asked to register some details for the exam (question 1) and to answer a multiple-choice question about their reason for participating in the exam (question 2). In the first survey, the students were also asked to rate their interest in each of the four specialties (ENT, ophthalmology, dermatology and plastic surgery) on a scale of 1 (poor) to 10 (high). This was asked as 4 separate questions, each corresponding to one of the four specialties, and each of the 4 questions was accompanied by a question asking the students to rate their knowledge in the specialty addressed “on a scale of 1(poor) to 10 (high)” (questions 3–10). The purpose of the 8 ‘rating’ questions was to gain an appreciation of the students’ baseline levels of interest and knowledge in the specialties involved, for comparison with levels after participation in the exam.

In the second survey, the participants were asked whether their participation in the exam was beneficial (question 1), they were asked to rate their interest and knowledge in the four specialties exactly as in the first survey (questions 2–9) and the last question was a free-text question about their opinions on the project (question 10).

The collected data was analysed using SPSS© v24 software and an independent samples t-test was performed to compare pre-exam and post-exam data. In order to avoid confounding results with data from the students who completed the pre-exam questionnaire but did not participate in the exam, only data from students who completed the pre-exam questionnaire, took the exam and completed the post-exam questionnaire was analysed in the comparison of pre- and post-exam levels of interest and knowledge in the EPOD specialties. This meant that data from 9 students who completed the first survey but did not participate in the exam was excluded from the analysis. It should be noted that all student responses to the question on motivation for participating in the exam were included, regardless of whether the student participated or not, because the question helps aid understanding into student motivation and the question is unrelated to whether the intervention affected students’ levels of interest and knowledge.

## Results

The initial questionnaire (the entrance survey) was completed by 25 students. Following registration details, the respondents were asked about their personal motivation for entering the prize exam.

The strongest motivating factor for participation was revision and examination technique (76% of respondents), followed by improvement of knowledge of the specialties (16%). Two of the students (8%) stated that they were interested in pursuing careers in one of these specialties and when they were given the further option to specify which specialty in the questionnaire, both stated ‘ophthalmology’. None of the students admitted to entering the Prize for the possibility of winning the prizes. The prizes were a £200 cash prize for the first prize and one textbook (from an EPOD specialty of choice) each for the second and third prize. All 16 students who actually attended for the exam received a certificate of participation regardless of exam outcome.

The response rate to the second questionnaire (the exit survey) was 94% (15/16). All 15 respondents felt that participation in the Prize Exam was beneficial for their learning. In order to determine whether the intervention (the exam) had resulted in higher levels of interest and knowledge in the specialties included, survey responses from the 16 exam participants were compared with the 15 responses received from the second survey (1 exam participant failed to respond to the post-exam survey). It should be noted that the number of participants in the exam dropped from 25 who registered for the exam to 16 who took part, due to non-attendance and last-minute drop-outs (apologies were sent to the lead for the exam).

Overall, compared to the pre-exam data, participating medical students attributed higher scores to knowledge and interest across all 4 specialties following the exam. See Tables 1 and 2.

**Table 1:** Differences in interest scores before and after exam

Interest scores (mean)			
	Before exam	After exam	Difference
**ENT**	5.5	6.1	+ 0.6
**Plastic Surgery**	4.6	4.9	+ 0.3
**Ophthalmology**	5.4	5.7	+ 0.3
**Dermatology**	4.2	5.4	+ 1.2

**Table 2:** Difference in knowledge scores before and after exam

Knowledge scores (mean)			
	Before exam	After exam	Difference
**ENT**	3.9	6.4	+ 2.5
**Plastic Surgery**	1.8	4.9	+ 3.1
**Ophthalmology**	4.0	5.7	+ 1.7
**Dermatology**	4.1	6.1	+ 2.0

The differences in Table 1 were not found to be statistically significant at the 95% confidence level. Although the students did attribute higher scores to interest after the exam, this was not statistically significant: ENT (*p* < 0.424), plastic surgery (*p* < 0.739), ophthalmology (*p* < 0.729) and dermatology (*p* < 0.199).

The statistical analysis demonstrated that differences in knowledge scores were statistically significant with a confidence level of 95% in terms of knowledge in ENT (*p* < 0.000), plastic surgery (*p* < 0.000), ophthalmology (*p* < 0.028) and dermatology (*p* < 0.012). In this cohort, ENT appears to be the preferred specialty (attracting the highest interest scores, pre- and post-exam) and the specialty, in which the students perceive that their knowledge is highest.

Additionally, respondents were also encouraged to provide free-text comments (see Additional file 1, student responses). Thematic analysis of the written feedback identified six common themes through coding (with some student quotes): ‘satisfaction’ (“an overall excellent pilot project”, “excellent pilot project”), ‘worthwhile experience’ (“great learning experience, enjoyed the exam and the feedback session”, “glad I did it” and “a valuable learning experience and one I’m very glad I got to partake in”), ‘useful revision’ (“the exam was very relevant to undergraduate outcomes and helped identify areas I hadn’t covered”), ‘high quality exam’ (“very high standard of questions which really pushed your knowledge” and “the questions were fair”), ‘timely feedback’ (“it was particularly good to get feedback straight afterwards, including explainations [sic] of each of the answers”,) and ‘expansion beneficial to students’ (“should definitely carry on and expand to bigger cohort group” and “there are a number of scholarship exams and I think this would be a great additional one”).

## Discussion

Our results show variable levels of interest in the EPOD specialties, however most students attributed low to moderate scores to this domain. This may reflect the subspecialist (perhaps niche) nature of these surgical specialties, however we reviewed papers relating to medical students’ career choices in ENT, ophthalmology, dermatology and plastic surgery, to understand whether the lack of exposure (and thereby, fewer opportunities to cultivate an interest) plays a role. A Japanese study about the career choices of senior medical students and foundation year doctors in 14 specialties demonstrated that, in most cases, career choice was strongly influenced by one of five factors. The five factors were categorised as “fulfilling life with job security”, “bioscientific orientation”, “advice from others”, “educational experience” and “personal reasons”. The findings showed that those who selected ophthalmology and dermatology were strongly correlated with wanting a “fulfilling life with job security”, whereas those who selected ENT were likely to have had similar influence from all 5 factors and thus, ENT alongside general medicine/family medicine was categorised as an intermediate group [12].

### ENT

The findings pertaining to the multi-factorial influence on a decision to pursue a career in ENT, highlighted by Takeda et al., are consistent with a UK study on medical students, foundation doctors and core surgical trainees applying for ENT national recruitment, which demonstrated that the most important factors for choosing a career in ENT were the variety of operative procedures, work–life balance, inherent interest in this clinical area and inspirational senior role models [13]. Bhutta et al. also found that exposure to ENT was strongly associated with pursuing a career in ENT [13]. A recent systematic review of factors affecting ENT as a career choice identified exposure to ENT as the most significant influential factor in medical students and junior doctors decisions’ to pursue it as a career [14].

### Plastic surgery

There is a shortage of literature on career choices in plastic surgery among UK and international graduates. This may be a potent area for further research in medical education by plastic surgeons or other parties such as the Royal College of Surgeons (RCS) or BAPRAS (British Association of Plastic and Reconstructive Surgery).

### Ophthalmology

Similar to dermatology, Takeda et al. found that a career choice in ophthalmology appealed to individuals who want a “fulfilling life with job security” [12]. In the UK, the Royal College of Ophthalmologists is pro-active in encouraging medical students to take an interest in ophthalmology. They hold a number of courses for undergraduates and run specialty prizes for medical students (e.g. the Duke-Elder prize and the Trevor-Roper Travel award). The Duke-Elder Prize in Ophthalmology is an official national prize exam for medical students which is organized by the Royal College of Ophthalmologists annually. Two studies have reviewed the impact of participation in the Duke-Elder Prize on pursuing ophthalmology as a career. One study found that participants in the Duke-Elder exam were not more likely to enter ophthalmology trainee than non-participants and that the presence of an ophthalmology undergraduate society at a medical school was correlated with a 1.37-fold increase of .29.5% of students who scored in the top 20 nationally in the Duke-Elder exam subsequently entered ophthalmology training [15]. Both studies concluded that, although the Duke-Elder exam is used as part of shortlisting criteria for ophthalmology training, there may be a perceived ‘unimportance’ of the exam in comparison to other criteria and this may dissuade students from participating in the exam [2, 15].

### Dermatology

Despite dermatology being one of the most competitive postgraduate specialties in the UK, several papers suggest that most dermatology career choices are made after qualification and experience during foundation and SHO jobs [7]. One study on British medical graduates found that dermatology was one of 5 specialties (alongside paediatrics, emergency medicine, radiology and cardiothoracic surgery), in which there was a statistically significant correlation (*p* < 0.001) between completing an undergraduate attachment and having an interest in pursuing a career in the specialty [16].

The findings of our literature review emphasize the importance of exposure to cultivating interest in a specialty and therefore we think smaller specialties such as the EPOD specialties should not be overlooked in medical education. As discussed in the introduction, the low amount of exposure to these specialties has been demonstrated to have implications on medical graduates’ knowledge and ability to manage associated conditions. This may compromise clinical care and patient safety if inadequate support and supervision is provided. Some research has been undertaken on the role of short courses in supplementing undergraduate education provided in these specialties and enhancing medical students’ interest. Short courses in both ENT [17] and plastic surgery [18] have been shown to have a positive impact on students’ career interests. Additionally, a transatlantic research group trialled public engagement lectures in dermatology for prospective medical students [19]. Such measures were considered valuable to students.

## Conclusions

All students reported that participation was beneficial to their learning. Based on our findings from the above data, the students enjoyed participation in the prize exam and it was useful for their revision and helped focus their studies for finals. They also learnt more about ENT, ophthalmology, dermatology and plastic surgery, which they perceive as being taught less in their curriculum as opposed to other specialties. Although there were not great increases in specific interests, this was a limited cohort used for a pilot study. Building on its success we intend on expanding this event to the entire stage 5-year group. This will enable greater participation in what is an additional voluntary event; inevitably helping those who have already or are yet to cultivate an interest in these specialties gather evidence early in their career of not only personal motivation but interest in and commitment to specialty.

It was encouraging to observe statistically significant increases in the students’ levels of knowledge in the EPOD specialties following the EPOD Prize Exam, as well as affirmative feedback on the project; however we recognise that perceived knowledge and increased confidence are not the same as true ability and that this is a limitation of our work. We note that there are aspects of knowledge (such as changes in attitudes and behaviours as result of educational experiences) that cannot be measured objectively. We chose to focus on whether students’ perceived that their knowledge and interest levels increased as this would indicate whether the project had educational and personal value to them.

Another limitation of our work is the small number of students (16) that took part in this pilot project. We attribute this small proportion of the cohort to a number of factors including timing of the exam, proximity to medical final exams and self-selection of students. Self-selection of students is a particularly important consideration as this may result in self-selection bias. Despite the possibility of self-selection bias, we think that our sample is representative of the cohort under the study as all students in the cohort were given the same opportunities to participate and no one was excluded. The impact of self-selection may be that the sample could be skewed towards higher academic ability or increased levels of enthusiasm for studies or greater capacity for self-directed learning; however the marks of the MCQ paper varied greatly indicating that students demonstrating different levels of academic ability (in the subjects examined) participated.

## Supplementary information

**Additional file 1.** Student Responses. Knowledge and interest scores pre and post exam. Data analysis.

## Data Availability

All data generated or analysed during this study are included in this published article [and its supplementary information files].

## Abbreviations

BAD: British Association of Dermatologists
BAPRAS: British Association of Plastic and Reconstructive Surgery
ENT: Ear, nose and throat surgery
EPOD: ENT, plastic surgery, ophthalmology and dermatology
GP: General Practice
MOSLER: Modified objective structured long examination review, a form of assessment used at Newcastle University Medical School
MSCAA: Medical Schools Council Assessment Alliance
RCS: Royal College of Surgeons
SBA: Single best answer

## References

[1] Hill S, Dennick R, Amoaku W (2017). Present and future of the undergraduate ophthalmology curriculum: a survey of UK medical schools. Int J Med Educ.

[2] Hsiao AM, Tatham AJ (2018). Factors at medical school influencing students’ decisions to pursue a career in ophthalmology. Eye (Lond).

[3] Baylis O, Murray PI, Dayan M (2011). Undergraduate ophthalmology education - a survey of UK medical schools. Med Teach.

[4] Powell J, Cooles FA, Carrie S, Paleri V (2011). Is undergraduate medical education working for ENT surgery? A survey of UK medical school graduates. J Laryngol Otol.

[5] Davis CR, O'Donoghue JM, McPhail J, Green AR (2010). How to improve plastic surgery knowledge, skills and career interest in undergraduates in one day. J Plast Reconstr Aesthet Surg.

[6] Clamp PJ, Gunasekaran S, Pothier DD, Saunders MW (2007). ENT in general practice: training, experience and referral rates. J Laryngol Otol.

[7] Barat A, Goldacre MJ, Lambert TW (2018). Career choices and career progression of junior doctors in dermatology: surveys of UK medical graduates. Dermatol Res Pract.

[8] Ah-Kee EY, Khan AA (2015). How can undergraduate ophthalmology teaching be improved?. Adv Med Educ Pract.

[9] Whitcroft KL, Moss B, McRae A (2016). ENT and airways in the emergency department: national survey of junior doctors’ knowledge and skills. J Laryngol Otol.

[10] Ferguson GR, Bacila IA, Swamy M (2016). Does current provision of undergraduate education prepare UK medical students in ENT? A systematic literature review. BMJ Open.

[11] Chiang YZ, Tan KT, Chiang YN, Burge SM, Griffiths CE, Verbov JL (2011). Evaluation of educational methods in dermatology and confidence levels: a national survey of UK medical students. Int J Dermatol.

[12] Takeda Y, Morio K, Snell L, Otaki J, Takahashi M, Kai I (2013). Characteristic profiles among students and junior doctors with specific career preferences. BMC Med Educ.

[13] Bhutta M, Mandavia R, Syed I (2016). A survey of how and why medical students and junior doctors choose a career in ENT surgery. J Laryngol Otol.

[14] Mayer AW, Smith KA, Carrie S. A systematic review of factors affecting choice of otolaryngology as a career in medical students and junior doctors. J Laryngol Otol. 2019:1–7. 10.1017/S0022215119001816 31535605

[15] Joshi L, Shanmuganathan VA, Kneebone RL, Amoaku W (2011). Performance in the Duke-elder ophthalmology undergraduate prize examination and future careers in ophthalmology. Eye (Lond).

[16] Ibrahim M, Fanshawe A, Patel V (2014). What factors influence British medical students’ career intentions?. Med Teach.

[17] Spiers H, Enayati H, Moussa R (2019). Augmenting ENT surgery outside the medical school curriculum: the role of a 1-day otolaryngology course. J Laryngol Otol.

[18] Spiers HVM, Zargaran A, Murtaza AN, Thomas A, Turki MAA, Ali F (2018). Enhancing medical curricula: the role of a 1-day plastic surgery course as an educational adjunct for medical students. J Surg Educ.

[19] Muthiah S, Wu K, Rajan N (2019). Public engagement lectures targeting prospective medical students: an opportunity for dermatology. Clin Exp Dermatol.

