# Supplementary material for: How does participation in a voluntary prize exam affect medical students’ knowledge and interest in ENT, plastic surgery, ophthalmology and dermatology?
Source: BMC Med Educ. 2020 Oct 27;20:387. doi: 10.1186/s12909-020-02314-y (PMC7592581; doi:10.1186/s12909-020-02314-y)
Supplement: Supplementary file 1 — Additional file 1. Student Responses. Knowledge and interest scores pre and post exam. Data analysis. [file 12909_2020_2314_MOESM1_ESM.zip › Appendix 1 Data analysisR1.pdf]

Appendix 1. Data analysis conducted with SPSS version 24

Group Statistics

	group	N	Mean	Std. Deviation	Std. Error Mean
ENT interest	Before exam	16	5.494	2.1230	.5308
	After exam	15	6.113	2.1334	.5508
ENT knowledge	Before exam	16	3.881	1.5398	.3849
	After exam	15	6.407	1.5457	.3991
Plastic surgery interest	Before exam	16	4.637	2.5783	.6446
	After exam	15	4.920	2.0512	.5296
Plastic surgery knowledge	Before exam	16	1.800	1.3266	.3317
	After exam	15	4.940	1.9838	.5122
Ophthalmology interest	Before exam	16	5.394	2.6624	.6656
	After exam	15	5.693	2.0440	.5278
Ophthalmology knowledge	Before exam	16	4.025	2.1980	.5495
	After exam	15	5.667	1.7220	.4446
Dermatology interest	Before exam	16	4.238	2.6475	.6619
	After exam	15	5.393	2.2053	.5694
Dermatology knowledge	Before exam	16	4.138	2.3298	.5824
	After exam	15	6.067	1.5578	.4022

Independent Samples Test

		Levene's Test for Equality of Variances		t-test for Equality of Means						
									95% Confidence Interval of the Difference	
		F	Sig.	t	df	Sig. (2-tailed)	Mean Difference	Std. Error Difference	Lower	Upper
ENT interest	Equal variances assumed	.016	.901	-.810	29	.424	-.6196	.7648	-2.1838	.9446
	Equal variances not assumed			-.810	28.852	.425	-.6196	.7649	-2.1844	.9452
ENT knowledge	Equal variances assumed	.006	.937	-4.555	29	.000	-2.5254	.5544	-3.6593	-1.3915
	Equal variances not assumed			-4.554	28.856	.000	-2.5254	.5545	-3.6597	-1.3911
Plastic surgery interest	Equal variances assumed	.848	.365	-.336	29	.739	-.2825	.8405	-2.0016	1.4366
	Equal variances not assumed			-.339	28.280	.737	-.2825	.8343	-1.9906	1.4256
Plastic surgery knowledge	Equal variances assumed	.539	.469	-5.212	29	.000	-3.1400	.6025	-4.3722	-1.9078
	Equal variances not assumed			-5.146	24.226	.000	-3.1400	.6102	-4.3988	-1.8812
Ophthalmology interest	Equal variances assumed	.952	.337	-.350	29	.729	-.2996	.8568	-2.0519	1.4528
	Equal variances not assumed			-.353	27.952	.727	-.2996	.8494	-2.0397	1.4406
Ophthalmology knowledge	Equal variances assumed	1.535	.225	-2.304	29	.029	-1.6417	.7125	-3.0989	-.1844
	Equal variances not assumed			-2.323	28.145	.028	-1.6417	.7069	-3.0893	-.1941
Dermatology interest	Equal variances assumed	.218	.644	-1.316	29	.199	-1.1558	.8784	-2.9523	.6407
	Equal variances not assumed			-1.324	28.622	.196	-1.1558	.8731	-2.9426	.6309
Dermatology knowledge	Equal variances assumed	15.029	.001	-2.691	29	.012	-1.9292	.7169	-3.3954	-.4629
	Equal variances not assumed			-2.725	26.308	.011	-1.9292	.7078	-3.3833	-.4750
