# Supplementary material for: How does participation in a voluntary prize exam affect medical students’ knowledge and interest in ENT, plastic surgery, ophthalmology and dermatology?
Source: BMC Med Educ. 2020 Oct 27;20:387. doi: 10.1186/s12909-020-02314-y (PMC7592581; doi:10.1186/s12909-020-02314-y)
Supplement: Supplementary file 1 — Additional file 1. Student Responses. Knowledge and interest scores pre and post exam. Data analysis. [file 12909_2020_2314_MOESM1_ESM.zip › Appendix knowledge and interest scores pre and post examR1.pdf]

Table 1. Knowledge and Interest levels before EPOD exam

	**ENT-I 1**	ENT-K 1	**PLA-I 1**	PLA-K 1	**OPH-I 1**	OPH-K 1	**DER-I 1**	DER-K 1
A	5.0	2.0	5.0	2.0	5.0	2.0	7.0	6.5
B	5.9	4.2	5.5	2.0	10	7.6	1.4	2.7
C	4.9	3.1	5.9	0.5	2.1	2.9	0.4	1.5
D	7.9	7.3	4.5	4.5	7.9	7.8	5.2	6.0
E	7.0	6.0	5.0	3.0	7.5	7.5	3.0	7.0
F	6.0	2.5	5.0	0.8	2.0	2.0	0.5	1.5
G	9.5	5.0	9.5	3.0	8.0	3.0	9.5	2.0
H	7.0	5.3	7.6	1.5	6.0	6.1	3.7	5.9
I	4.9	2.7	1.9	0.7	5.3	1.2	4.1	1.3
J	4.0	3.0	3.0	2.0	6.0	5.0	5.0	6.0
K	1.1	1.4	0.1	0.1	1.7	2.5	0.9	1.3
L	7.0	3.9	3.4	1.1	3.9	3.0	4.1	2.4
M	3.6	4.2	0.3	0.3	4.1	2.7	4.4	6.2
N	7.0	3.1	7.7	2.1	8.5	4.1	7.9	6.2
O	4.7	4.8	6.3	4.2	6.8	4.9	6.5	6.9
P	2.4	3.6	3.5	1.0	1.5	2.1	4.2	2.8
AVERAGE	5.5	3.9	4.6	1.8	5.4	5.0	4.2	4.1

Table 2. Knowledge and interest levels after EPOD exam

Respondent	**ENT-I 2**	ENT-K 2	**PLA-I 2**	PLA-K 2	**OPH-I 2**	OPH-K 2	**DER-I 2**	DER-K 2
1	6.4	4.6	3.2	5.1	5.4	3.4	2.9	4.3
2	3.8	6.5	6.2	4.5	3.1	4.7	1.0	3.4
3	6.5	8.5	6.5	6.7	7.0	8.3	7.9	8.2
4	2.6	4.9	1.0	3.6	7.0	4.9	1.8	4.9
5	8.2	7.5	7.2	8.5	8.2	7.8	7.8	8.1
6	1.4	2.7	0.6	0.0	2.7	2.3	5.6	4.3
7	7.2	8.0	5.4	5.3	2.6	4.9	5.0	8.0
8	8.9	5.3	6.1	6.1	7.7	6.5	7.0	4.7
9	6.0	8.0	4.0	5.0	7.0	7.5	4.0	5.5
10	4.8	7.1	4.7	6.8	4.4	5.3	5.1	5.4
11	7.0	7.5	5.0	6.0	7.5	7.5	5.0	7.0
12	8.0	7.0	5.0	5.0	4.0	6.0	7.0	6.0
13	6.0	6.0	5.0	2.5	6.0	4.0	6.5	7.0
14	8.0	6.4	6.0	4.0	8.5	6.6	8.3	6.7
15	6.9	6.1	7.9	5.0	4.3	5.3	6.0	7.5
AVERAGE	6.1	6.4	4.9	4.9	5.7	5.7	5.4	6.1

*Note that one respondent did not participate in the second survey.
