# Supplementary material for: How does participation in a voluntary prize exam affect medical students’ knowledge and interest in ENT, plastic surgery, ophthalmology and dermatology?
Source: BMC Med Educ. 2020 Oct 27;20:387. doi: 10.1186/s12909-020-02314-y (PMC7592581; doi:10.1186/s12909-020-02314-y)
Supplement: Supplementary file 1 — Additional file 1. Student Responses. Knowledge and interest scores pre and post exam. Data analysis. [file 12909_2020_2314_MOESM1_ESM.zip › Appendix Student ResponsesR1.pdf]

Question 10: What did you think about this project? (and is this worthwhile repeating on a larger scale?) Any reflections on how the project has affected you (e.g. knowledge, revision, future career choice) are much appreciated. Please provide as much feedback as possible. We are hoping to make this project official and would like to extend this to the other base units.

Responses:

Student 1: “Really enjoyed the exam - good questions and cases appropriate for testing knowledge. Learnt a lot from it, would liked [sic] to have participated in the clinical exam for mosler practice and prep.”

Student 2: “Really useful- it covered knowledge and skills that I want to develop for the finals exam. The teaching session was useful as knowing what I’d got wrong and why was very helpful to my learning. Doing the Mosler was beneficial as I was able to practice doing a challenging Mosler station. think it’s a really good idea to expand it to other base units.”

Student 3: “An overall excellent project. Would have liked to have Urology teaching and questions but that is personal preference. All teaching fellows were excellent, questions balanced from core to merit style well. Would revise some of the wording on two ENT questions. 1. The Monospot test was worded 'what investigation must they receive before being discharged' which confused me. The presentation was clearly a tonsillitis/mono so I assumed it was a two part question as it related to discharge and so picked HIV as a potential precipitant to EBV infection. Changing the phrasing to the more commonly used 'what would be the most appropriate investigation?' would be far better. Could even include GI exam as an option. 2. The thyroid lump options should be clearer regarding the ultrasound and 2WW. Maybe options should change to 'Two Week Wait Referral to ENT Clinic' and 'Arrange an Outpatient Ultrasound'. I accept that one option was 'more correct' but it could have been clearer.”

Student 4: “Very high standard of question which really pushed your knowledge. I found going through the paper’s answers very useful. There are a number of scholarship exams and I think this one would be a great additional one.”

Student 5: “Excellent pilot project, well organised. Should definitely carry on and expanding to bigger cohort group.”

Student 6: “I really liked the idea of the EPOD exam, the questions were fair and I liked how we get teaching and certificates afterwards for everyone, that was one of the most appealing aspects for me. I think that it would be improved by having more questions, and therefore more teaching, and the Powerpoint slides available afterwards for revision? Also, some teaching on how to do a Mosler in that speciality would be useful too, but overall, a great idea and glad I did it! Thank you.”

Student 7: “The written paper was a useful way to assess my knowledge prior to finals. It was particularly good to get feedback straight afterwards, including explainations [sic] of each of the answers. The practical exam was a good opportunity to practise moslers in ENT, dermatology and ophthalmology. I received some really useful constructive feedback about what I did well and how I could improve. In the future, it would be good if all students participating in the written exam could also have the opportunity to do the practise moslers.”

Student 8: “More things like this please!”

Student 9: “Helpful exam. Learnt a lot!”

Student 10: “Thought it was a great idea- useful for revision but also quite interesting as well.”

Student 11: “The exam was very relevant to undergraduate outcomes and helped identify areas that I hadn’t covered.”

Student 12: “Great learning experience, enjoyed the exam and the feedback session.”

Student 13: “Useful to practice questions on these subjects and have the answered discussed afterwards. Interesting to learn more about these specialities.”

Student 14: “I thought this was a great way to help gain knowledge before our exams. I'm personally not interested in any of the four specialties as future careers, however this was a valuable learning opportunity and one I'm very glad I got to partake in. Many thanks for organising this!”

Student 15: “It was a great opportunity to touch on specialties we rarely cover at medical school. Really useful idea for the students who want to pursue a career in one of the specialities.”
